# Recurrent hepatitis C treatment with direct acting antivirals – a real life study at a Brazilian liver transplant center

**DOI:** 10.1590/1414-431X20198519

**Published:** 2019-08-05

**Authors:** L.P. Zanaga, A.G. Santos, E.C. Ataíde, I.F.S.F. Boin, R.S.B. Stucchi

**Affiliations:** 1 Disciplina de Infectologia, Departamento de Clínica Médica, Faculdade de Ciências Médicas, Universidade Estadual de Campinas, Campinas, SP, Brasil; 2 Grupo de Fígado, Hipertensão Portal e Transplante Hepático, Disciplina de Moléstias do Aparelho Digestivo, Departamento de Cirurgia, Faculdade de Ciências Médicas, Universidade Estadual de Campinas, Campinas, SP, Brasil

**Keywords:** Hepatitis C, Liver transplantation, Direct acting antivirals, Sofosbuvir, Daclatasvir, Recurrent hepatitis

## Abstract

Recurrent hepatitis C (HCV) after liver transplantation (LT) is an important cause of morbidity and mortality. Antiviral treatment is recommended to avoid unfavorable outcomes. Direct-acting antivirals (DAA) have transformed HCV treatment, with higher efficacy and fewer side-effects than interferon-based therapies traditionally used. To evaluate DAA treatment outcomes at a Brazilian transplant unit, data of patients who finished HCV treatment at the Liver Transplant Unit of the University of Campinas were analyzed. Treatment consisted of sofosbuvir, daclatasvir, and ribavirin, for 12 or 24 weeks, according to the national guidelines. Fifty-five patients completed antiviral treatment and 54 had HCV-viral load results available. The majority of patients were male (78%), 58 years old on average, 65% had hepatocellular carcinoma (HCC) before LT, and 67% were interferon treatment-experienced. Most patients had HCV genotype 1 (65%), 35% had genotype 3, and started treatment on an average of 38 months after LT (range: 2–228). Fifty-eight percent were treated for 12 weeks and 42% for 24 weeks, using a mean dose of ribavirin of 10.1 mg/kg (4.2–16.1). There were no treatment interruptions due to serious side effects. The sustained virological response rate was 98%. Only one patient relapsed, a genotype 3 cirrhotic treated for 12 weeks. The average follow-up after starting antivirals was 20 months. There were no recurrences of HCC, but there was one rejection episode and one cirrhosis decompensation episode, both 12 weeks after treatment. DAA treatment is safe and effective in the post-LT setting and was not associated to HCC recurrence in the cohort studied.

## Introduction

Hepatitis C virus (HCV) is the main cause of liver transplantation (LT) worldwide and has universal recurrence (1,2). If untreated, it can lead to cirrhosis and graft loss in one third of patients within five years of LT, which justifies the reduced survival rates observed when comparing HCV to other causes of LT (2). The progression of liver fibrosis is accelerated in the post-LT population (3) and its treatment is considered a priority, since antiviral therapy can be used to prevent liver damage and, consequently, prolong survival (4–6). The achievement of sustained virological response (SVR) is related to both histological and clinical improvement (4,6). Therapeutic options, once dependent on interferon and reaching 30% SVR rates, are now based on direct acting antivirals (DAA), drugs with better tolerability, reduced side effects, and optimized response rates, with overall SVR rates post-LT higher than 90% (7–14). The optimal drug regimen, duration, and timing of treatment are the issues faced by physicians in the DAA era.

## Material and Methods

This was a retrospective observational study performed at the Liver Transplant Unit of the University of Campinas.

### Patients

The study included patients who were at least 18 years old, submitted to deceased-donor orthotopic liver transplantation due to complications of chronic hepatitis C infection (end-stage liver cirrhosis or hepatocellular carcinoma) with positive HCV viral load (HCV-VL), who started antiviral treatment with DAA from December 1st 2015 until December 31st 2017.

The exclusion criteria were: severe chronic kidney disease (defined by glomerular filtration rate <30 mL·min^-1^·(1.73 m^2^)^-1^, calculated with the MDRD formula), active neoplasia, anemia (hemoglobin levels <10 g/dL), arrhythmia, and severe or decompensated clinical comorbidities (defined as concomitant diseases that could lead to poor treatment adherence or treatment discontinuation, such as decompensated diabetes, recent stroke, unstable angina, and ischemic heart disease).

### Antiviral therapy

Antiviral treatment consisted of the combination of 400 mg sofosbuvir (SOF) daily, 60 mg daclatasvir (DCV) daily, and ribavirin (RBV, doses ranging from 250 to 1250 mg daily), for 12 or 24 weeks, according to the Brazilian hepatitis C treatment guidelines published in 2015 (15).

Ribavirin doses were at the discretion of the attending physician and could be reduced in case of hemolytic anemia. Erythropoietin could be used in doses up to 40,000 IU weekly, especially for symptomatic patients with hemoglobin levels <10 g/dL. Safety was evaluated in all patients at least monthly during treatment and at four and 12 weeks after the end of treatment (EOT).

Antiviral efficacy was assessed with the detection of HCV-VL at baseline, at weeks 4, 12, and 24 weeks of treatment, and at 12 and 24 weeks after treatment. Serum HCV-RNA viral loads were measured with the Abbott Real Time HCV Test (USA) with a lower limit of quantification of 12 IU/mL. Rapid virological response was characterized by undetectable HCV-VL at treatment week 4 and SVR was defined as undetectable HCV-VL 12 weeks after EOT.

### Immunosuppression

Immunosuppression was managed according to the local guidelines (16), usually consisting of corticosteroids (generally withdrawn within 6 months after LT) and a calcineurin inhibitor as the main immunosuppressive agent (cyclosporine or tacrolimus), at times associated to mycophenolate. The main immunosuppressive agent was not changed to start antiviral treatment. Patients on prednisone had its dose tapered, if possible, at the beginning of antiviral treatment and mycophenolate was discontinued whenever possible, due to increased risk of anemia when associated with RBV.

### Data collection

Data regarding demographic, clinical, virological, and treatment-related variables were collected from the patient records using a standardized form.

### Statistical analysis

Continuous variables are reported as medians and ranges and categorical data as percentages. A P value of <0.05 was considered significant. All analyses were performed using EpiInfo 7.1.5.2 (CDC, USA). All patients who fulfilled the inclusion criteria and received at least one dose of medication during the study period were included in the safety statistical analysis. Efficacy was assessed in all patients who completed treatment and had HCV-VL tested 12 weeks after EOT.

### Ethical considerations

The study was approved by the local Ethics Committee. A standardized informed consent issued by the Brazilian Ministry of Health was obtained for each patient who started antiviral treatment, in accordance with the 2015 Brazilian hepatitis C treatment guidelines.

## Results

From December 1st 2015 to December 31st 2017, 55 patients received HCV antiviral treatment at the Liver Transplant Unit of the State University of Campinas and fulfilled the inclusion criteria for the study. One patient was excluded due to chronic kidney disease with a glomerular filtration rate (GFR) of less than 30 mL·min^-1^·(1.73m^2^)^-1^ and one patient was excluded because he was diagnosed with metastatic squamous cell carcinoma and discontinued antiviral treatment.

### Demographic and baseline characteristics

Most patients were male (78%), with a median age of 58 years (Table 1). The majority of patients (36 patients, 65%) had HCV genotype 1 and 35% had genotype 3 infection. There were no infections by genotypes 2, 4, 5, or 6. Most patients were interferon treatment-experienced (37 patients, 67%), either before (53%) or after LT (25%). All patients were DAA naive. Median baseline viral load was 1,315,445 IU/mL (range: 2,297–18,500,000).

**Table 1:** Patient demographics and baseline characteristics.

Variable	n = 55
Age, years, median (range)	58 (40−71)
Gender, n (%)	
Male	43 (78)
Female	12 (22)
HCV genotype, n (%)	
1	1 (2)
1A	21 (38)
1B	14 (25)
3	19 (35)
Previous treatment, n (%)	
Naive	18 (33)
Interferon-experienced	37 (67)
Interferon-experienced, n (%)	
Before LT	29 (53)
After LT	14 (25)
Anemia, n (%)	12 (22)
Chronic kidney disease, n (%)	13 (24)
Diabetes mellitus, n (%)	25 (45)
History of HCC, n (%)	36 (65)
Immunosuppression, n (%)	
Tacrolimus	40 (73)
Cyclosporine	9 (16)
Azathioprine	2 (4)
Everolimus	12 (22)
Sirolimus	2 (4)
Mycophenolate	10 (11)
Prednisone	9 (16)
None	1 (2)

HCV: hepatitis C virus; LT: liver transplantation; HCC: hepatocellular carcinoma.

Fibrosis staging prior to antiviral therapy was not routinely performed. According to the national treatment guidelines (15), post-LT patients should be treated regardless of the fibrosis level, and staging information is not required for access to treatment. Among the patients studied, liver biopsy was performed only when there was evidence of elevated transaminases, in order to differentiate HCV inflammatory activity from graft rejection. Twenty-three patients (42%) had been biopsied during the last two years before treatment: 26% had mild fibrosis (Metavir F1), 43% had moderate fibrosis (F2), 9% F3, and 4% F4. Three biopsies (13%) had histological signs of steatosis and three patients had features of acute rejection (which had already been managed by the moment antiviral treatment was started).

Thirteen patients (24%) had chronic kidney disease, with GFR between 30–60 mL·min^-1^·(1.73m^2^)^-1^ and 25 patients (45%) had diabetes mellitus. Two patients had undergone liver and kidney transplantation. Personal history of hepatocellular carcinoma (HCC) before LT was present in 36 patients (65%). No patients had HIV or hepatitis B coinfection.

Immunosuppression was managed according to local guidelines and most patients were on tacrolimus- (40 patients, 73%) and/or everolimus-based regimens (12 patients, 22%). One patient did not use any immunosuppressive medication due to chimaerism.

### Antiviral treatment characteristics

Interferon-free antiviral treatment was started at a median of 38 months after LT (Table 2) and most patients (58%) were treated for 12 weeks with a combination of SOF, DCV, and RBV. The most common dosage of RBV was 750 mg daily (64% patients), with a median of 10.1 mg/kg body weight.

**Table 2:** Antiviral treatment characteristics.

Variable	n = 55
Time from LT to treatment, months, n (range)	38 (2−228)
Duration, n (%)	
12 weeks	32 (58)
24 weeks	23 (42)
RBV dose at baseline, n (%)	
250 mg	1 (2)
500 mg	5 (9)
750 mg	34 (62)
1000 mg	8 (14)
1250 mg	7 (13)
RBV dose at baseline, mg/kg, n (range)	10.1 (4.2-16.1)
HCV-VL at baseline, IU/mL, median (range)	1,315,445 (2,297−18,500,000)
Undetectable HCV-VL at week 4, n (%)	28 (51)

LT: liver transplantation; RBV: ribavirin; HCV: hepatitis C virus; VL: viral load.

### Antiviral treatment side effects

Antiviral treatment was generally safe and well tolerated. Anemia was the most common side effect (65%), with 11 patients (20%) reaching hemoglobin levels under 10 g/dL. Forty-five percent of patients required RBV dose reduction, 11% used erythropoietin, and 3 received blood transfusions for management of anemia. Hyperbilirubinemia (characterized by serum bilirubin levels over 2 mg/dL) occurred in 18% of patients, without need for any intervention.

There were no treatment interruptions due to DAA-related adverse events. Only one patient required treatment interruption, at week 11, because he was diagnosed with disseminated *Mycobacterium tuberculosis* infection and the concomitant use of SOF/DCV and rifampicin is contraindicated due to deleterious drug interactions.

There was no diagnosis of rejection during antiviral treatment. Twenty (36%) patients underwent changes in the immunosuppressive medication, mostly tapering and suspension of prednisone or mycophenolate (Table 3).

**Table 3:** Adverse effects of antiviral treatment.

Variable	n = 55
Anemia	
Hb<12.5 g/dL	36 (65%)
Hb<10.0 g/dL	11 (20%)
Hyperbilirubinemia	10 (18%)
Management of adverse events	
Reduction of RBV dose	25 (45%)
Suspension of RBV	3 (5%)
Erythropoietin use	6 (11%)
Rejection during DAA treatment	0
Change in immunosuppressive medication	20 (36%)
Tapering of prednisone	6 (11%)
Reduction of mycophenolate	1 (2%)
Suspension of mycophenolate	4 (7%)
Switch from EVR to FK	2 (4%)
Reduction of FK dosage	4 (7%)
Increase of FK dosage	4 (7%)

Data are reported as n and percent within parentheses. Hb: hemoglobin; RBV: ribavirin; DAA: direct-acting antiviral; FK: tacrolimus; EVR: everolimus.

### Treatment outcomes

Fifty-one percent of treated patients had a rapid virological response (RVR), with undetectable HCV-VL at week 4. The SVR rate observed was 98% (53/54). One patient was lost to follow-up after EOT and there was no HCV-VL result available to evaluate virological response.

There was only one treatment relapse, diagnosed at week 4 post-treatment. The patient was a 61-year-old Caucasian woman, with HCV genotype 3 infection and Child-Pugh A cirrhosis, previously interferon-experienced. The patient was treated with SOF/DCV/RBV for 12 weeks, according to the Brazilian guidelines at that moment. The patient began treatment using RBV 750 mg daily, 7.7 mg/kg, but had its dose reduced and eventually suspended its use at the eighth week due to severe anemia. The baseline HCV-VL was 1,315,445 IU/mL (6.12 log). The patient had undetectable HCV-VL at treatment week 4 (RVR), but relapsed at weeks 4 and 12 post-treatment, when HCV-VL results were, respectively, 9,470,000 IU/mL (log 6.98) and 24,800,000 IU/mL (log 7.4).

There is a concern regarding potential kidney toxicity for patients treated with SOF. Among the 54 patients who finished treatment and had SVR12 results the median glomerular filtration rate (GFR) was 71.6 mL·min^-1^·(1.73m^2^)^-1^ at the beginning of treatment and 74 mL·min^-1^·(1.73m^2^)^-1^ 12 weeks after EOT. Both patients with kidney and liver transplantation had stable GFR during and after antiviral treatment.

The median follow-up period after the beginning of treatment was 20 months (range: 10–31). There was no diagnosis of recurrence of HCC since the beginning of antiviral treatment. Among the study population there was one rejection episode detected 12 weeks after EOT, managed with adjustment of tacrolimus dosage. One patient had cirrhosis decompensation, with esophageal variceal bleeding, ascites, encephalopathy, and jaundice occurring twelve weeks after EOT (Table 4).

**Table 4:** Antiviral treatment outcomes.

Variable	n = 54
SVR, n (%)	53 (98%)
Follow-up, months, n (range)	20 (10−31)
HCC recurrence, n (%)	0%
Rejection after DAA treatment, n (%)	1 (2%)

SVR: sustained virological response; HCC: hepatocellular carcinoma; DAA: direct-acting antiviral.

## Discussion

This real-life study in Brazilian liver transplant recipients confirmed the results of international clinical trials, demonstrating the dramatic improvement in SVR rates that DAA therapy has brought compared to traditional interferon-based therapy. SOF, an NS5B inhibitor, and DCV, an NS5A inhibitor, were among the first DAA available for interferon-free treatment worldwide.

The Brazilian hepatitis C guidelines published in 2015 (15) recommended the use of SOF associated to DCV or simeprevir, with or without RBV to treat patients with recurrent HCV. The attending physicians at the Liver Transplant Unit opted for treatment of all patients with DCV, instead of simeprevir, due to fewer drug-drug interactions with immunosuppressants (17,18).

The ALLY-1 trial evaluated 53 post-LT patients treated with SOF, DCV, and RBV for 12 weeks (9). Ribavirin was used at a lower dosage (480 mg on average) than in the present study and five patients discontinued the drug due to side effects. The overall SVR rate was similar to the current study, 94% (50/53 patients), 95% among genotype 1 patients (39/41), and 91% (10/11) among genotype 3 patients. The HCV-Target study also evaluated the use of SOF and DCV, with or without RBV, with a similar overall SVR rate of 96.6% among LT patients (10 [Bibr B11] [Bibr B12] [Bibr B13]). Another Brazilian cohort of 39 patients treated with SOF and DCV, with or without RBV, for 12 weeks, reached an overall SVR rate of 89.7%. (19). All treatment failures were genotype 3-infected patients, similar to the present study. A German real-life study including ten patients treated with SOF and DCV, with or without RBV, achieved 100% SVR (20).

Favorable results can also be obtained with other DAA combinations, such as ombitasvir/paritaprevir/ritonavir and dasabuvir/ledipasvir/SOF or simeprevir and SOF, with or without RBV. The SVR rates achieved in clinical studies range from 80 to 100% (10–14,20).

The high treatment response rates are accompanied by relatively low frequencies of adverse events, which can be managed with less difficulties (8–10). In our study, there were no treatment discontinuations due to adverse effects and the literature shows reduced rates of treatment discontinuation, ranging from 1.7–2% (9,10). Suspension of ribavirin due to anemia was 5% in the present study and 8% in the ALLY-1 study (9).

The recurrence of HCC, an important cause of concern (21), was not observed in the population studied, concurring with several papers in the literature (22–27), but in contrast to reports of non-LT patients (28–31) that raised concern of the possibility of HCC induced by DAA-therapy. Conti et al. (28) evaluated 344 patients treated with DAA and after 24 weeks of follow-up it was observed that 7.6% of patients had HCC, affecting 28.8% of patients with and 3% of those without previous HCC.

Another unique factor involving LT patients is the occurrence of graft rejection. The rates observed in the present study with DAA were dramatically lower than a previous study at the same institution with interferon-based therapy (0 *vs* 48.6% of patients treated, with 38.9% of the rejection episodes considered to be related to interferon therapy) (32). The only rejection case (2% of the 55 patients included) was diagnosed 12 weeks after EOT. This result is comparable to recent reports, with graft rejection rates ranging from 0 to 4.2% (10,18,21). The European Liver and Intestine Transplant Association recommends careful monitoring of immunosuppression levels during treatment and particularly after EOT, since improved liver function can alter exposure to immunosuppressants (33).

In Brazil, HCV treatment is funded and provided by the Ministry of Health. The real-life treatment experience of Brazilian patients is of paramount importance to evaluate the success of public health policies and the need for incorporation of other therapeutic possibilities.

Limitations of this study include its observational nature and small sample size, which hamper analyses of factors associated with treatment failure and the role of ribavirin for achieving SVR. The presence of universal fibrosis staging prior to HCV treatment could allow analyses of the relationships between fibrosis and virological response and adverse effects. Even though it is not necessary for treatment recommendation, the knowledge of the fibrosis staging can be helpful in order to determine which patients would benefit from the use of RBV or 24 weeks of treatment, not to mention its role in establishing the need for HCC surveillance. Tests for resistance-associated variants could have been performed to help analyze if the treatment failure observed was due to antiviral resistance, which could influence the choice of subsequent treatment for the patient. The short follow-up after antiviral treatment did not allow any considerations on the relationship between DAA therapy and HCC recurrence. Further studies with larger populations and longer observation periods are necessary to clarify this matter.

## References

[1] Razavi H, Waked I, Sarrazin C, Myers RP, Idilman R, Calinas F (2014). The present and future disease burden of hepatitis C virus (HCV) infection with today's treatment paradigm. J Viral Hepat.

[2] Firpi RJ, Clark V, Soldevila-Pico C, Morelli G, Cabrera R, Levy C (2009). The natural history of hepatitis C cirrhosis after liver transplantation. Liver Transpl.

[3] Berenguer M, Schuppan D (2013). Progression of liver fibrosis in post-transplant hepatitis C: mechanisms, assessment and treatment. J Hepatol.

[4] Picciotto FP, Tritto G, Lanza AG, Addario L, De Luca M, Di Costanzo GG (2007). Sustained virological response to antiviral therapy reduces mortality in HCV reinfection after liver transplantation. J Hepatol.

[B05] Tanaka T, Selzner N, Therapondos G, Renner EL, Lilly LB (2013). Virological response for recurrent hepatitis C improves long-term survival in liver transplant recipients. Transpl Int.

[6] Bizollon T, Pradat P, Mabrut JY, Chevallier M, Adham M, Radenne S (2005). Benefit of sustained virological response to combination therapy on graft survival of liver transplanted patients with recurrent chronic hepatitis C. Am J Transplant.

[7] Charlton M, Everson GT, Flamm SL, Kumar P, Landis C, Brown RS (2015). Ledipasvir and sofosbuvir plus ribavirin for treatment of HCV infection in patients with advanced liver disease. Gastroenterology.

[8] Faisal N, Bilodeau M, Aljudaibi B, Hirsch G, Yoshida EM, Hussaini T (2016). Sofosbuvir-based antiviral therapy is highly effective in recurrent hepatitis C in liver transplant recipients: Canadian multicenter "real-life" experience. Transplantation.

[9] Poordad F, Schiff ER, Vierling JM, Landis C, Fontana RJ, Yang R (2016). Daclatasvir with sofosbuvir and ribavirin for hepatitis C virus infection with advanced cirrhosis or post-liver transplantation recurrence. Hepatology.

[10] Saxena V, Khungar V, Verna EC, Levitsky J, Brown RS, Hassan MA (2017). Safety and efficacy of current direct-acting antiviral regimens in kidney and liver transplant recipients with hepatitis C: results from the HCV-Target study. Hepatology.

[B11] Kwo PY, Mantry PS, Coakley E, Te HS, Vargas HE, Brown R (2014). An interferon-free antiviral regimen for HCV after liver transplantation. N Engl J Med.

[B12] Pungpapong S, Aqel B, Leise M, Werner KT, Murphy JL, Henry TM (2015). Multicenter experience using simeprevir and sofosbuvir with or without ribavirin to treat hepatitis C genotype 1 after liver transplant. Hepatology.

[B13] Manns M, Samuel D, Gane EJ, Mutimer D, McCaughan G, Buti M (2016). Ledipasvir and sofosbuvir plus ribavirin in patients with genotype 1 or 4 hepatitis C virus infection and advanced liver disease: a multicentre, open-label, randomized, phase 2 trial. Lancet Infect Dis.

[14] Gutierrez JA, Carrion AF, Avalos D, O'Brien C, Martin P, Bhamidimarri KA (2015). Sofosbuvir and simeprevir for treatment of hepatitis C virus infection in liver transplant recipients. Liver Transpl.

[15] BRASIL (2015). Ministério da Saúde. Secretaria de Vigilância em Saúde. Departamento de DST, AIDS e Hepatites Virais. Protocolo clínico e diretrizes terapêuticas para hepatite C e coinfecções. Brasília, DF.

[16] Hospital de Clínicas da UNICAMP (2012). Manual de processos de trabalho do transplante de fígado. Campinas.

[17] Tisher S, Fontana RJ (2014). Drug-drug interactions with oral anti-HCV agents and idiosyncratic hepatotoxicity in the liver transplant setting. J Hepatol.

[18] Ouwerkerk-Mahadevan S, Snoeys J, Peeters M, Beumont-Mauviel M, Simion A (2016). Drug-drug interactions with the NS3/4A protease inhibitor simeprevir. Clin Pharmacokin.

[19] Mucenic M, Brandão ABM, Marroni CA, Fleck AM, Zanotelli ML, Kiss G (2018). Daclatasvir and sofosbuvir with or without ribavirin in liver transplant recipients: a single-center real-world study. Transpl Proc.

[20] Rupp C, Hippchen T, Neuberger M, Sauer P, Pfeiffenberger J, Stremmel W (2018). Successful combination of direct antiviral agents in liver-transplanted patients with recurrent hepatitis C virus. World J Gastroenterol.

[21] Sanaka S, Karasala GR, Tillman HL (2018). A downside to hepatitis C virus cure? Vigilance is needed regarding hepatitis B virus reactivation, organ rejection, or hepatocellular carcinoma progression. J Infect Dis.

[22] Adhoute X, Penaranda G, Raoul JL, Sellier F, Castellani P, Oules V (2018). Hepatocellular carcinoma recurrence in hepatitis C virus-related cirrhosis treated with direct-acting antivirals: a case-control study. Eur J Gastroenterol Hepatol.

[B23] Bielen R, Moreno C, Van Vlierberghe H, Bourgeois S, Mulkay JP, Vanwolleghem T (2017). The risk of early occurrence and recurrence of hepatocellular carcinoma in hepatitis C infected patients treated with direct acting antivirals with and without pegylated interferon: a Belgian experience. J Viral Hepat.

[B24] Cabibbo G, Petta S, Calvaruso V, Cacciola I, Cannavò MR, Madonia S (2017). Is early recurrence of hepatocellular carcinoma in HCV cirrhotic patients affected by treatment with direct-acting antivirals? A prospective multicentre study. Aliment Pharmacol Ther.

[B25] Cammè C, Cabibbo G, Craxì A (2016). Direct antiviral agents and risk for HCC early recurrence: much ado about nothing. J Hepatol.

[B26] Ikeda K, Kawamura Y, Kobayashi M, Kominami Y, Fujiyama S, Sezaki H (2017). Direct-acting antivirals decreased tumor recurrence after initial treatment of hepatitis C virus-related hepatocellular carcinoma. Dig Dis Sci.

[27] ARNS collaborative study group on hepatocellular carcinoma (2016). Lack of evidence of an effect of direct acting antivirals on the recurrence of hepatocellular carcinoma. J Hepatol.

[28] Conti F, Buonfiglioli F, Scuteri A, Crespi C, Bolondi L, Caraceni P (2016). Early occurrence and recurrence of hepatocellular carcinoma in HCV-related cirrhosis treated with direct acting anitivirals. J Hepat.

[B29] Reig M, Marião Z, Perelló C, Iãarrairaegui M, Ribeiro A, Lens S (2016). Unexpected early tumor recurrence in patients with hepatitis C virus-related hepatocellular carcinoma undergoing interferon-free therapy. J Hepat.

[B30] El Kassas M, Funk AL, Salaheldin M, Shimakawa Y, Eltabbakh M, Jean K (2018). Increased recurrence rates of hepatocellular carcinoma after DAA therapy in a hepatitis C infected Egyptian cohort: a comparative analysis. J Viral Hepat.

[31] Warzyszynska K, Jonas M, Wasiak D, Kosieradzki M, Malkowski P (2017). Accelerated hepatocellular carcinoma recurrence rate after postoperative direct-acting antivirals treatment - preliminary report. Clin Exp Hepatol.

[32] Zanaga LP, Vigani AG, Angerami RN, Giorgetti A, Escanhoela CAF, Ataíde EC (2017). Survival benefits of interferon-based therapy in patients with recurrent hepatitis C after orthotopic liver transplantation. Braz J Med Biol Res.

[33] Belli L, Duvoux C, Berenguer M, Berg T, Coilly A, Colle I (2017). ELITA consensus statements on the use of DAAs in liver transplant candidates and recipients. J Hepat.

